# COVID-19 severity and in-hospital mortality in an area with high HIV prevalence

**DOI:** 10.4102/sajhivmed.v24i1.1412

**Published:** 2023-01-27

**Authors:** Michael T. Boswell, Tshegofatso Maimela, Dan Hameiri-Bowen, George Riley, Albertus Malan, Nickietta Steyn, Nomonde Nolutshungu, Talita R. de Villiers, Zelda de Beer, John Mathabathe, Khanyisile Tshabalala, Fareed Abdullah, Rajiev Ramlall, Marthinus Heystek, Debashis Basu, Paul Rheeder, Veronica Ueckermann, Wesley van Hougenhouck-Tulleken

**Affiliations:** 1 Department of Internal Medicine, Steve Biko Academic Hospital, University of Pretoria, Pretoria, South Africa; 2 Clinical Public Health Unit, Department of Public Health Medicine, Steve Biko Academic Hospital, Pretoria, South Africa; 3 Nuffield Department of Medicine, Oxford University, Oxford, United Kingdom; 4 Tshwane District Hospital, Pretoria, South Africa; 5 Department of Medical Immunology, University of Pretoria, Pretoria, South Africa; 6 South African Medical Research Council, Pretoria, South Africa

**Keywords:** HIV, COVID-19, SARS-CoV-2, mortality, biomarker

## Abstract

**Background:**

HIV infection causes immune dysregulation affecting T-cell and monocyte function, which may alter coronavirus disease 2019 (COVID-19) pathophysiology.

**Objectives:**

We investigated the associations among clinical phenotypes, laboratory biomarkers, and hospitalisation outcomes in a cohort of people hospitalised with COVID-19 in a high HIV prevalence area.

**Method:**

We conducted a prospective observational cohort study in Tshwane, South Africa. Respiratory disease severity was quantified using the respiratory oxygenation score. Analysed biomarkers included inflammatory and coagulation biomarkers, CD4 T-cell counts, and HIV-1 viral loads (HIVVL).

**Results:**

The analysis included 558 patients, of whom 21.7% died during admission. The mean age was 54 years. A total of 82 participants were HIV-positive. People living with HIV (PLWH) were younger (mean age 46 years) than HIV-negative people; most were on antiretroviral treatment with a suppressed HIVVL (72%) and the median CD4 count was 159 (interquartile range: 66–397) cells/µL. After adjusting for age, HIV was not associated with increased risk of mortality during hospitalisation (age-adjusted hazard ratio = 1.1, 95% confidence interval: 0.6–2.0). Inflammatory biomarker levels were similar in PLWH and HIV-negative patients. Detectable HIVVL was associated with less severe respiratory disease. In PLWH, mortality was associated with higher levels of inflammatory biomarkers. Opportunistic infections, and other risk factors for severe COVID-19, were common in PLWH who died.

**Conclusion:**

PLWH were not at increased risk of mortality and those with detectable HIVVL had less severe respiratory disease than those with suppressed HIVVL.

**What this study adds:**

This study advances our understanding of severe COVID-19 in PLWH.

## Introduction

People living with HIV (PLWH) have an increased risk of mortality from infection with respiratory viruses, including influenza and human metapneumovirus.^1,2^ Many studies have reported that PLWH, especially those not on antiretroviral treatment (ART) and with a detectable HIV-1 viral load (HIVVL), have a higher risk of coronavirus disease 2019 (COVID-19)-related in-hospital mortality.^3,4,5^ However, observational cohort studies of hospitalised patients with COVID-19 have reported that PLWH had lower oxygen requirements during their admission. In these studies, patients with detectable HIVVL had lower relative risk of intubation than PLWH with suppressed HIVVL.^6,7^ In an observational study, PLWH had similar outcomes after initiation of high-flow nasal oxygen or ventilatory support.^8^ There is still some uncertainty as to whether COVID-19 immunopathology and clinical phenotypes are altered by HIV coinfection.

Current evidence suggests that severe COVID-19 is associated with dysregulation of the monocyte-macrophage response, defective T-cell responses, elevated inflammatory cytokines, and hyperactivated neutrophils which culminate in ongoing, inappropriate systemic inflammation which damages pulmonary and other tissues.^9,10,11,12^ People hospitalised with COVID-19, particularly with the more severe spectrum of disease, can develop acute respiratory distress syndrome (ARDS), which is associated with systemic inflammation.^13^ Established markers of respiratory disease severity in COVID-19 include respiratory oxygenation (ROX) scores and partial pressure of oxygen (PaO_2_)/fraction of inspired oxygen (FIO_2_) ratios.^8,13,14^ Antiviral therapy in early COVID-19 leads to lower risk of hospitalisation and mortality.^15,16,17^ In addition, anti-inflammatories with either broad and non-specific targets, like high-dose corticosteroids, or targeted, like the interleukin 6 inhibitor tocilizumab, also lead to reduced risk of in-hospital mortality.^18^

HIV infection has strong effects on cellular immune phenotypes and function, affecting T and B lymphocytes, and monocytes – all of which are implicated in COVID-19 pathophysiology.^19,20,21^ Recently, more evidence has emerged to assess the effect of HIV on cellular immune responses on COVID-19. HIV coinfection does not appear to alter severe acute respiratory syndrome coronavirus type 2 (SARS-CoV-2) CD4+ function or phenotypes, but is associated with reduced CXCR3 expression on CD8+ T-cells.^22,23^ Higher HIVVLs are also associated with increased expression of activation markers on CD8+ T-cells in COVID-19, which may alter disease phenotypes. People living with HIV mount similar SARS-CoV-2-specific antibody responses in acute COVID-19 to HIV-negative people.^24^ HIV viraemia alters monocyte subpopulation phenotypes, reducing CCR2 and CX3CR1 expression, which may affect their ability to move from blood into tissue. In COVID-19 this may reduce pulmonary inflammation.^21^

The majority of PLWH analysed in larger cohorts or systematic reviews were on ART, with suppressed HIVVL and high CD4 T-cell counts.^3,4,25^ Consequently, the effect of HIV viraemia and associated immunological changes on the clinical manifestations of COVID-19 remain poorly described. Uncertainty also exists as to the extent to which comorbidities in PLWH, such as hypertension, diabetes, or opportunistic infections, contribute to the increased risk of morbidity and mortality.

We investigated whether HIV infection is associated with COVID-19 severity, differences in routinely collected laboratory biomarkers, and in-hospital mortality in a well-characterised clinical cohort of patients admitted with COVID-19, in a setting with a high HIV prevalence.

## Methods

### Cohort description

We conducted a prospective, single centre, observational cohort study of patients admitted to the Tshwane District Hospital complex (TDH) from April 2020 to November 2020. All patients were admitted with COVID-19 as determined by a positive SARS-CoV-2 PCR test. This period encompasses the first COVID-19 wave in Pretoria and predates the widespread prevalence of the SARS-CoV-2 Beta/B.1.351 variant which occurred during the second wave from December 2020 to February 2021. This hospital complex was the primary referral centre for COVID-19 cases in the greater Tshwane area, encompassing a population of approximately 3 000 000 people, with a HIV prevalence of 10.5% (95% confidence interval [CI]: 7.7% – 14.1%) in 2017.^26,27^ The hospital could admit 66 adults to general ward care levels, a dedicated COVID-19 High Care (HC) unit (22 adult beds) and an intensive care unit (ICU) (10 adult beds). The HC unit admitted patients in need of dialysis, close monitoring on oxygen, high-flow nasal oxygen and non-invasive ventilation. The ICU was used for patients who were intubated and ventilated.

Data including demographic information, comorbidities, presence of central obesity (classified as ‘overweight’), date of admission, symptom onset, date of SARS-CoV-2 PCR test, vital signs, level of care, and admission outcome were captured on standardised case report forms (CRF). The CRFs were completed by treating clinicians during patients’ admission. Data from the CRFs were entered by research assistants into a REDCap database hosted by the University of Pretoria and reviewed for accuracy by clinicians involved in the study.^28^ Hospital admission outcome was coded as survived or died. ‘Survived’ included patients transferred to other hospitals for further medical care after discharge from the COVID-19 units. ‘Died’ included patients with confirmed deaths during hospitalisation for COVID-19. We analysed age as a continuous variable and additionally stratified the cohort into age groups with 20 year-increments.

**FIGURE 1:**
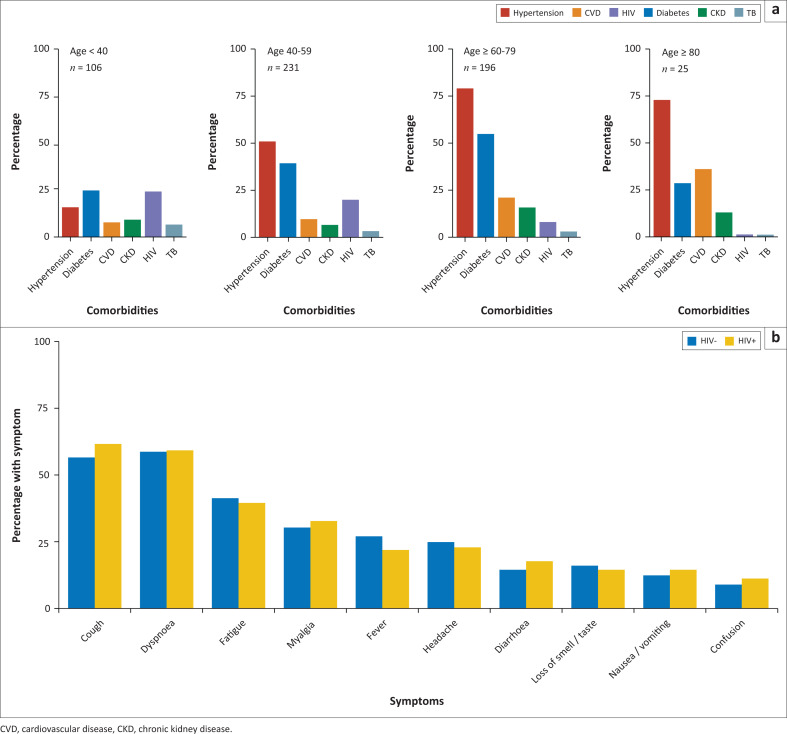
(a) The prevalence of comorbidities by age categories is shown. HIV and diabetes were the most common comorbidities in patients younger than 40 years. Prevalence of non-communicable comorbidities increased with age, and HIV prevalence decreased. (b) No significant differences in symptoms at admission by HIV status (chi-squared *P* > 0.05 for all pairwise comparisons). Cough and dyspnoea were the most common symptoms at admission.

Admission vital sign data were taken as the worst score within a 48 h window around the date of admission (admission ± 24 h). The ROX score was calculated for participants with admission vital data (Online Appendix 1 Figure 1-A1).^14^ The ROX score is a continuous variable which estimates respiratory disease severity by creating a composite score considering the supplemental oxygen concentration, peripheral oxygen saturation, and respiratory rate.

Laboratory biomarker data were extracted from the South African National Health Laboratory Services (NHLS) online data warehouse. We analysed haematology panels (full blood count; differential white cell counts including absolute neutrophil count [ANC], lymphocyte count [ALC] and neutrophil-to-lymphocyte ratio [NLR]); organ function biomarkers (creatinine, alanine aminotransferase [ALT]); inflammatory biomarkers (C-reactive protein [CRP], ferritin, procalcitonin [PCT]); and D-dimer (DDIM). Laboratory biomarkers were aggregated as median values for admission ± 24 h. We analysed CD4+ T-cell counts taken during admission. CD4+ T-cell counts were stratified into higher (equal to, or above, 200 cells/µL) and lower (below 200 cells/µL) CD4 counts. Plasma HIVVL was assessed from the previous 12 months and during admission, with the most recent value being used for this analysis. HIV-1 viral loads were stratified at 1000 copies/mL into detectable (equal to, or above, 1000 copies/mL) and suppressed (below 1000 copies/mL).

### Statistical analysis

Data were analysed in R Studio.^29,30,31,32,33^ Baseline characteristics of participants were summarised as means and standard deviations, medians and interquartile ranges (IQR), and counts with percentages as appropriate. Pairwise comparisons of continuous variables were done using Mann-Whitney (MW) U tests or *T* tests, dependent on the variables’ distribution. Comparison of proportions was performed using chi-squared tests, or Fisher’s exact test (FET). Correlations were analysed by Spearman Rank or Pearson correlation coefficients – depending on the variable’s distribution. The sensitivity and specificity of variables’ ability to predict higher levels of supportive care were calculated using Area Under the Receiver Operating Characteristic curve (AUROC). An AUROC cut-off of 0.7 was used to decide if a variable had good predictive ability for a specified outcome.

The association between age and comorbidities was analysed by logistic regression models, with age treated as a continuous variable, and results reported as odds ratios (OR). Univariate survival analysis was analysed by Kaplan-Meier survival curves with hypothesis testing via log rank tests. Multivariable survival analysis was done using Cox regression models, with results reported as hazard ratios (HR). Schoenfield residuals were used to test for violation of the proportional hazard’s assumption. Markers of disease severity, including ROX scores, and laboratory biomarkers were used to stratify the cohort into mutually exclusive tertiles of comparable size.

### Ethical considerations

Ethics approval was granted by the University of Pretoria’s Faculty of Health Sciences Research Ethics Committee, and permission obtained from the institutional authorities to collect clinical data from patients admitted to the Tshwane District Hospital complex. Protocol ethics reference number: 637/2020.

## Results

### Cohort description

A total of 558 patient records were analysed (Table 1). The mean age of this cohort was 54 (standard deviation [s.d.] ± 16) years with equivalent numbers of male (50.5%) and female (49.5%) patients. Hypertension and diabetes were the most common comorbidities, at 55% and 41%. Younger patients were more likely to be HIV positive at admission (age and HIV: odds ration [OR] = 0.96, 95% confidence interval [CI]: 0.94–0.97, *P* < 0.001), and older patients were more likely to have non-communicable comorbidities including hypertension (OR = 1.07, 95% CI: 1.06–1.09, *P* < 0.001), diabetes (OR = 1.03, 95% CI: 1.02–1.04, *P* < 0.001), cardiovascular disease (OR = 1.04, 95% CI: 1.03–1.06, *P* < 0.001) and chronic kidney disease (OR = 1.02, 95% CI: 1.0–1.04, *P* = 0.03) (Figure 1).

**TABLE 1:** Cohort demographics, admission vital signs and biomarker levels.

COVID-19 hospital cohort (*N* = 558)	Mean	s.d.	*n*	%	Median	IQR
Age in years	54	16	-	-	-	-
Male	-	-	282	50.5	-	-
Hypertension	-	-	304	54.5	-	-
Diabetes	-	-	227	40.7	-	-
Cardiovascular disease	-	-	75	13.4	-	-
Chronic kidney disease	-	-	54	9.7	-	-
Cancer in past 5 years	-	-	13	2.3	-	-
Overweight	-	-	124	22.2	-	-
General ward	-	-	418	75.9	-	-
High Care	-	-	85	15.4	-	-
ICU	-	-	48	8.7	-	-
Died	-	-	121	21.9	-	-
Received steroids	-	-	432	78.0	-	-
HIV-positive	-	-	82	14.7	-	-
Past TB diagnosis	-	-	25	4.5	-	-
Active TB diagnosis	-	-	14	2.5	-	-
Days hospitalised	-	-	-	-	6.00	3–10
Respiratory rate bpm	-	-	-	-	22.00	20–25
Estimated FiO_2_ %	-	-	-	-	0.37	0.21–0.70
Peripheral O_2_ saturation %	-	-	-	-	93.00	90–96
ROX score	-	-	-	-	8.20	4.8–16.7
Systolic blood pressure mm/Hg	-	-	-	-	123.00	111–136
ANC × 10^−9^/L	-	-	-	-	6.70	4.8–10.0
ALC × 10^−9^/L	-	-	-	-	1.10	0.8–1.6
NLR	-	-	-	-	6.20	3.4–9.8
CRP mg/L	-	-	-	-	108.00	46–181
Ferritin µg/L	-	-	-	-	527.00	238–1171
PCT µg/L	-	-	-	-	0.13	0.05–0.58
DDIM mg/L	-	-	-	-	0.88	0.39–1.95
HbA1c %	-	-	-	-	7.20	6.3–10.3
Creatinine µmol/L	-	-	-	-	84.00	67–115
ALT U/L	-	-	-	-	32.00	20–54

Note: Vital signs and laboratory biomarker levels measured at admission (±24 h).

s.d., standard deviation; IQR, interquartile range; bpm, beats per minute. FiO_2_, fraction of inspired oxygen; ROX, respiratory oxygenation score; ANC, absolute neutrophil count; ALC, absolute lymphocyte count; NLR, neutrophil: lymphocyte ratio; CRP, C-reactive protein; PCT, procalcitonin; DDIM, D-dimer; ALT, alanine aminotransferase; TB, tuberculosis; ICU, intensive care unit; HbA1c%, haemoglobin A1c percentage.

**FIGURE 2:**
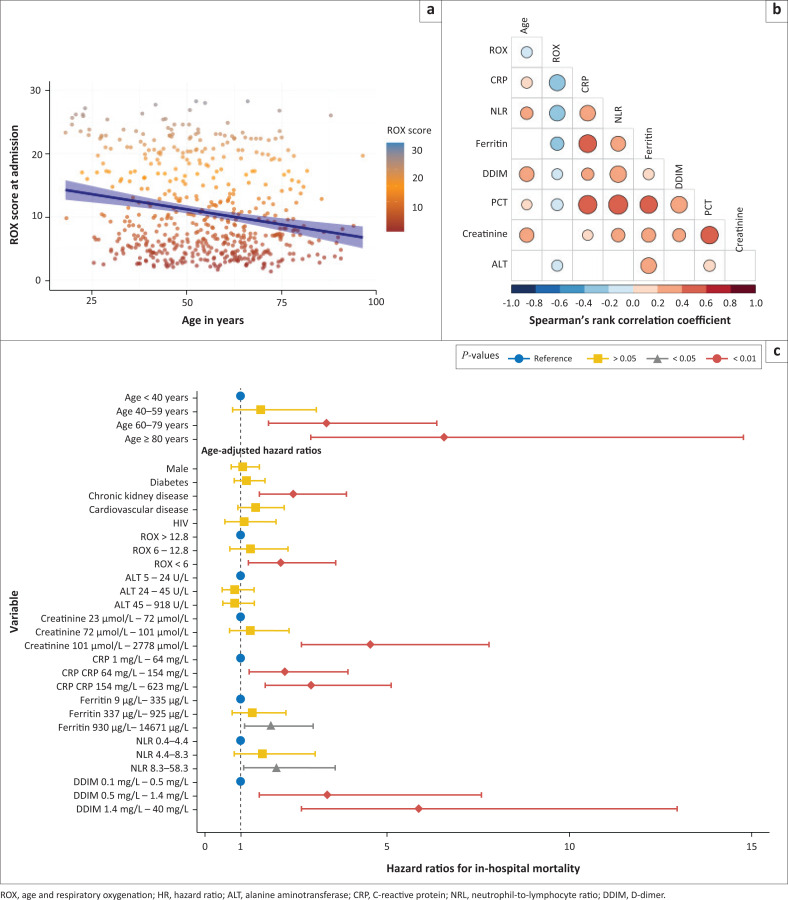
(a) Age and respiratory oxygenation (ROX) scores at admission correlated negatively (rho = –0.2, *P* < 0.001). (b) Correlation matrix of biomarkers with age and ROX score, non-significant correlations are shown as blank cells. The size and colour of the circles show the strength and direction of the Spearman correlation coefficients. (c) Forest plot of the hazard ratio (HR) and their confidence intervals for variables association with in-hospital mortality. Age was associated with a strong effect on mortality; therefore, all other HRs are age-adjusted.

A total of 82 PLWH were admitted during this period (15% of cohort). People living with HIV were younger than HIV-negative patients and were less likely to have hypertension or cardiovascular disease (CVD) (Table 2). People living with HIV were more likely to have a previous, or current, diagnosis of tuberculosis (TB), but the number of patients with active TB was small (*n* = 14). CD4 counts were available for 56 (68.3%) patients, and the median CD4 count was 159 (IQR: 66–397) cells/µL. A total of 32/56 (61.5%) PLWH had CD4 counts below 200 cells/µL. HIV-1 viral loads were available for 52 (63.4%), and the median HIVVL was 59 789 (IQR: 9417–194 534) copies/mL. HIV-1 viral loads were suppressed in 37/52 (71.2%). The median duration of symptoms before admission was slightly longer in PLWH (8.4 days vs 7.1 days, *P* = 0.04). There was no difference in the proportion of symptoms reported at admission, with cough and dyspnoea the most common (Figure 2) (chi-squared *P* > 0.05 for all comparisons).

**TABLE 2:** Summary and comparison of admission variables by HIV status.

Variable	HIV-negative (*n* = 476)						PLWH (*n* = 82)						*P*
	Mean	s.d.	*n*	%	Median	IQR	Mean	s.d.	*n*	%	Median	IQR	
Age in years	56	±16	-	-	-	-	46	±12			-	-	**< 0.001**
Male	-	-	243	51.1	-	-	-	-	39	47.6	-	-	0.630
Hypertension	-	-	275	57.8	-	-	-	-	29	35.4	-	-	**< 0.001**
Diabetes	-	-	200	42.0	-	-	-	-	27	32.9	-	-	0.140
Cardiovascular disease	-	-	73	15.3	-	-	-	-	2	2.4	-	-	**0.001**
Chronic kidney disease	-	-	47	9.9	-	-	-	-	7	8.5	-	-	0.840
Cancer in past 5 years	-	-	10	2.1	-	-	-	-	3	3.7	-	-	0.420
Overweight	-	-	111	23.3	-	-	-	-	13	15.9	-	-	0.150
General ward	-	-	354	75.3	-	-	-	-	64	79.0	-	-	0.200
High Care	-	-	71	15.1	-	-	-	-	14	17.3	-	-	0.200
ICU	-	-	45	9.5	-	-	-	-	3	3.7	-	-	0.200
Died	-	-	109	23.1	-	-	-	-	12	14.8	-	-	0.110
Received steroids	-	-	372	78.2	-	-	-	-	60	74.0	-	-	0.310
Previous TB diagnosis	-	-	7	1.5	-	-	-	-	18	22.0	-	-	**< 0.001**
Active TB diagnosis	-	-	5	1.1	-	-	-	-	9	11.0	-	-	**< 0.001**
Days hospitalised	-	-	-	-	6	3–10	-	-	-	-	6.00	3–10	0.730
Respiratory rate bpm	-	-	-	-	22	20–25	-	-	-	-	22.00	18–25	0.850
Estimated FiO_2_	-	-	-	-	0.40	0.21–0.70	-	-	-	-	0.22	0.21–0.58	0.070
Peripheral O_2_ saturation	-	-	-	-	93	90–95	-	-	-	-	94.00	91–96	**0.020**
ROX score	-	-	-	-	7.9	4.7–15.9	-	-	-	-	9.80	5.5–19.5	0.210
Systolic blood pressure mm/Hg	-	-	-	-	124	112–137	-	-	-	-	118.00	105–130	**0.001**
ANC × 10^−9^/L	-	-	-	-	7.0	4.4–10.5	-	-	-	-	6.20	4.2–9.2	0.180
ALC × 10^−9^/L	-	-	-	-	1.2	0.8–1.6	-	-	-	-	1.10	0.7–1.6	0.540
NLR	-	-	-	-	6.2	3.4–10.3	-	-	-	-	6.00	3.5–9.2	0.810
CRP mg/L	-	-	-	-	108	47–181	-	-	-	-	110.00	42–183	0.990
Ferritin µg/L	-	-	-	-	520	232–1232	-	-	-	-	542.00	295–1028	0.870
PCT µg/L	-	-	-	-	0.14	0.05–0.59	-	-	-	-	0.08	0.04–0.46	0.330
DDIM mg/L	-	-	-	-	0.88	0.39–1.95	-	-	-	-	1.03	0.36–2.08	0.860
HbA1c %	-	-	-	-	7.1	6.3–10.3	-	-	-	-	7.70	6.2–12.4	0.390
Creatinine µmol/L	-	-	-	-	85	68–115	-	-	-	-	78.00	61–103	**0.030**
ALT U/L	-	-	-	-	33	20–54	-	-	-	-	31.00	19–47	0.400

Note: Vital signs and biomarker levels measured at admission (±24 h). *P*-values in bold significance tested at < 0.05 shown for Fisher’s exact test for categorical variables, *T* test (age comparison) and MW test (all other continuous variables).

PLWH, People living with HIV; s.d., standard deviation; *N*, sample size; IQR, interquartile range; FiO_2_, fraction of inspired oxygen; ROX, respiratory oxygenation score; ANC, absolute neutrophil count; ALC, absolute lymphocyte count; NLR, neutrophil: lymphocyte ratio; CRP, C-reactive protein; PCT, procalcitonin; DDIM, D-dimer; ALT, alanine aminotransferase; TB, tuberculosis; ICU, intensive care unit; HbA1c%, haemoglobin A1c percentage.

### Respiratory oxygenation scores and laboratory biomarkers are associated with COVID-19 severity and mortality

The median ROX score at admission was 8.2 (IQR: 4.8–16.7). Increasing age was associated with lower ROX scores at admission (correlation of age and ROX score: rho = –0.2, *P* < 0.001), and higher DDIM and inflammatory biomarker levels (Figure 2a and Figure 2b). ROX scores, inflammatory biomarkers and DDIM levels showed significant collinearity with each other (Figure 2b).

A total of 85 patients required HC as their highest level of supportive care and 48 patients were admitted to ICU. Length of hospitalisation was longer for those admitted to ICU than the general wards (median 16 days vs 5 days, *P* < 0.001). In addition, patients admitted to ICU had more severe disease – ROX scores were lower, ANC, PCT, DDIM and ALT levels were significantly higher when compared to patients admitted to HC or general wards (Online Appendix 1 Table 1-A1). Consequently, higher ROX scores, ANC, NLR and PCT levels predicted admission to ICU with AUROC > 0.7. ROX scores below 4.5 had the highest specificity at 82% (sensitivity 62%), and PCT above 0.13 the highest sensitivity at 94% (specificity 57%). The mortality rate was 57% for patients admitted to ICU, 24% for HC, and 17% for general ward admissions (FET *P* < 0.001 for ICU vs HC or General wards).

**FIGURE 3:**
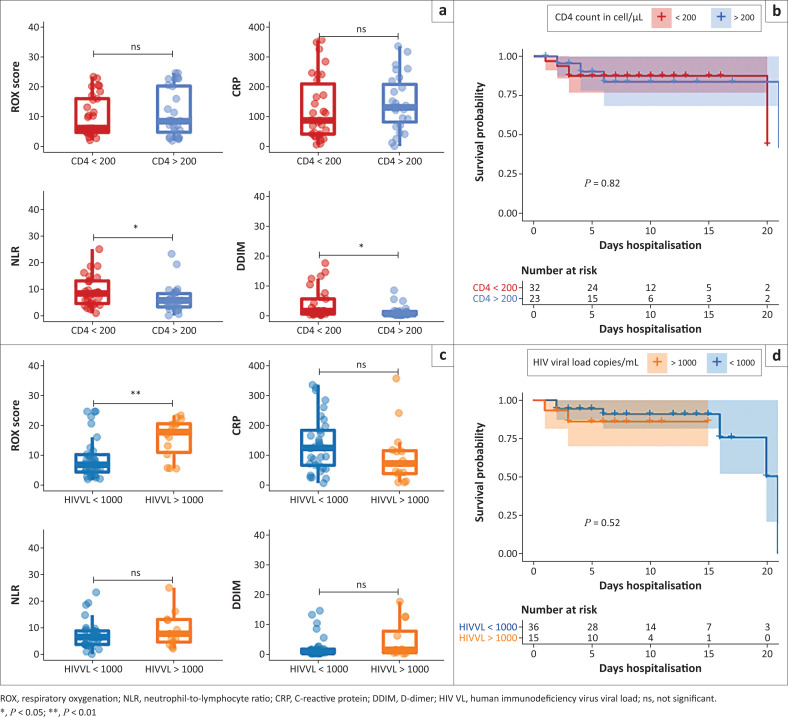
(a) Pairwise comparisons for Respiratory oxygenation (ROX) scores, C-reactive protein (CRP), neutrophil-to-lymphocyte ratio (NLR) and D-dimer (DDIM) levels are shown for higher and lower CD4 counts. Lower CD4 counts were associated with higher NLR and DDIM levels at admission. (b) Kaplan-Meier survival curves are shown for PLWH stratified by CD4 count. There was no significant difference in time to death in hospital, logrank *P*-value shown. (c) Pairwise comparisons for ROX scores, CRP, NLR and D-dimer levels are shown for PLWH by HIVVL. An HIVVL above 1000 copies/mL was associated with significantly higher ROX scores at admission. (d) Kaplan-Meier survival curves overlapped for these patients indicating no significant difference in survival, logrank *P*-value shown.

A total of 121 (21.7%) patients died during admission. Increasing age was associated with increased mortality during admission (Figure 2c). A ROX score below six at admission was associated with a twofold increase in mortality compared to higher ROX scores (age-adjusted hazard ratio [aHR] = 2.1, 95% CI: 1.2–3.6, *P* = 0.01). The tertiles grouping the highest levels of creatinine, CRP, ferritin, NLR and DDIM were also associated with increased mortality (Figure 3c). Gender and diabetes were not associated with increased mortality during hospitalisation, but chronic kidney disease was (aHR = 2.4, 95% CI: 1.5–3.9, *P* < 0.001). HIV was not associated with significantly increased mortality (aHR = 1.1, 95% CI: 0.6–2.0, *P* = 0.14) (Figure 2c).

### COVID-19 severity in people living with HIV

Respiratory rates were similar between PLWH and HIV-negative patients; however, PLWH needed less oxygen at admission, and had higher peripheral oxygen saturation readings. Respiratory oxygen scores were higher in PLWH, but the difference was not statistically significant (Table 2). There was also no significant difference in NLR, CRP, ferritin, PCT, DDIM, ALT, or haemoglobin A1c (HbA1c) between PLWH and HIV-negative patients. Creatinine levels were slightly lower in PLWH, when compared to HIV-negative patients. These associations were unchanged in linear regression models which adjusted for age differences in HIV-negative patients and PLWH. People living with HIV were as likely as HIV-negative patients to be admitted to ICU – 3.7% of PLWH versus 9.5% HIV-negative patients (FET *P* = 0.2).

### Markers of disease severity in people living with HIV, stratified by CD4 count and HIV-1 viral load

In PLWH, a CD4 count below 200 cells/µL was associated with lower odds of having hypertension, diabetes or being on ART (Online Appendix 1 Table 2-A1). People living with HIV with CD4 counts below 200 cells/µL had higher HIVVLs, NLRs and DDIM levels (Figure 3a) and were more likely to have TB (25% vs 4%, *P* = 0.06) when compared to PLWH with higher CD4 counts. Respiratory oxygen scores were equivalent between those with higher and lower CD4 counts (Figure 3a). Admission rates to HC or ICU were equivalent between PLWH with CD4 counts above or below 200 cells/µL.

People living with HIV with detectable HIVVLs were less likely to be on ART (OR = 0.11, 95% CI: 0.01–0.59, *P* = 0.003), and had lower median CD4 counts when compared to those with suppressed HIVVLs (34 [IQR: 16–47] vs 256 [IQR: 134–429], MW *P* < 0.0001) (Table 3). People living with HIV with detectable HIVVLs were younger than those with suppressed HIVVLs (mean age 40 years vs 48 years, *T* test *P* = 0.02) and were less likely to have diabetes (OR = 0.12, 95% CI: 0.003–0.97, *P* = 0.04). People living with HIV with detectable HIVVLs had significantly higher ROX scores than those with a suppressed HIVVL (17.8 [IQR: 10.9–20.5] vs 6.7 [IQR: 4.3–10.1], MW *P* = 0.005). Laboratory biomarker levels were not associated with HIVVL (Figure 3c). None of the PLWH with detectable HIVVLs was admitted to HC or ICU.

**TABLE 3:** Comparison of people with HIV by viral load.

Variable	HIVVL < 1000 (*n* = 37)						HIVVL > 1000 (*n* = 15)						*P*
	Mean	s.d.	*n*	%	Median	IQR	Mean	s.d.	*n*	%	Median	IQR	
Age in years	48	10	-	-	-	-	40.00	11			-	-	**0.020**
Male	-	-	15	40.5	-	-	-	-	8	53.3	-	-	0.540
Hypertension	-	-	18	48.6	-	-	-	-	3	20.0	-	-	**0.070**
Diabetes	-	-	14	37.8	-	-	-	-	1	6.7	-	-	**0.040**
Cardiovascular disease	-	-	1	2.7	-	-	-	-	1	6.7	-	-	0.490
Chronic kidney disease	-	-	4	10.8	-	-	-	-	0	0.0	-	-	0.310
Cancer in past 5 years	-	-	2	5.4	-	-	-	-	0	0.0	-	-	1.000
Overweight	-	-	9	24.3	-	-	-	-	1	6.7	-	-	0.250
Died	-	-	6	16.7	-	-	-	-	2	13.3	-	-	1.000
Received steroids	-	-	27	73.0	-	-	-	-	10	66.7	-	-	0.740
Antiretroviral therapy	-	-	34	91.9	-	-	-	-	8	53.3	-	-	**0.005**
CD4 count > 200	-	-	22	64.7	-	-	-	-	1	7.1	-	-	**< 0.001**
Previous TB	-	-	11	29.7	-	-	-	-	5	33.3	-	-	1.000
Active TB	-	-	1	2.7	-	-	-	-	6	40.0	-	-	**0.001**
Days hospitalised *n*	-	-	-	-	8.00	6–13	-	-	-	-	6.00	3–9	0.110
Respiratory rate bpm	-	-	-	-	24.00	18–28	-	-	-	-	20.00	18–22	0.140
Estimated FiO_2_	-	-	-	-	0.40	0.21–0.78	-	-	-	-	0.21	0.21–0.26	**0.006**
Peripheral O_2_ saturation	-	-	-	-	94.00	90–96	-	-	-	-	94.00	92–97	0.620
ROX score	-	-	-	-	6.77	4.34–10.14	-	-	-	-	17.72	10.92–20.50	**0.005**
Systolic blood pressure mm/Hg	-	-	-	-	119.00	105–130	-	-	-	-	117.00	92–123	0.280
ANC × 10^−9^/L	-	-	-	-	6.53	5.11–8.32	-	-	-	-	5.17	3.41, 7.82	0.350
ALC × 10^−9^/L	-	-	-	-	1.07	0.76–1.51	-	-	-	-	0.87	0.26, 1.54	0.170
NLR	-	-	-	-	6.47	3.68, 8.79	-	-	-	-	7.74	4.60, 13.12	0.350
CRP mg/L	-	-	-	-	124.00	66–183	-	-	-	-	72.00	38–114	**0.090**
Ferritin µg/L	-	-	-	-	548.00	291–982	-	-	-	-	365.00	309–969	0.740
PCT µg/L	-	-	-	-	0.06	0.04–0.25	-	-	-	-	0.08	0.06–5.94	0.310
DDIM mg/L	-	-	-	-	0.72	0.38–1.71	-	-	-	-	1.46	0.52–7.74	0.280
HbA1c %	-	-	-	-	8.30	6.40–12.30	-	-	-	-	6.20	6.10–9.70	0.540
Creatinine µmol/L	-	-	-	-	158.00	253	-	-	-	-	119.00	207	0.600
ALT U/L	-	-	-	-	40.08	32	-	-	-	-	34.67	22	0.570
CD4 count cells/µL	-	-	-	-	271.00	153–422	-	-	-	-	34.00	15–47	**< 0.001**
HIVVL IU/mL	-	-	-	-	-	-	-	-	-	-	59 789.00	9417–19 4534	-

Note: Vital signs and biomarker levels measured at admission (± 24 h); *P*-values in bold significance tested at < 0.05 shown for Fisher’s exact test for categorical variables, *T* test (age comparison) and Mann-Whitney test (all other continuous variables).

HIVVL, HIV-1 viral load; s.d., standard deviation; n, sample size; IQR, interquartile range; FiO_2_, fraction of inspired oxygen; ROX, respiratory oxygenation score; ANC, absolute neutrophil count; ALC, absolute lymphocyte count; NLR, neutrophil: lymphocyte ratio; CRP, C-reactive protein; PCT, procalcitonin; DDIM, D-dimer; ALT, alanine aminotransferase; TB, tuberculosis; HbA1c%, hemoglobin A1c percentage.

### Variables associated with mortality by HIV status

In total, 109/476 (23.1%) HIV-negative patients died during admission. Among HIV-negative patients, those who died were older (mean age 62 vs 52 years, *T* test *P* < 0.001), and more likely to have hypertension (*P* < 0.001), diabetes (*P* = 0.06), CVD (*P* < 0.001), or CKD (*P* < 0.001). Respiratory disease severity was significantly worse at admission in HIV-negative patients who died versus those who survived (ROX score: 4.8 vs 9.5, MW *P* < 0.001). Mortality in the HIV-negative patients was also associated with higher levels of laboratory biomarkers, higher ANC, lower ALC and higher creatinine levels (MW *P* < 0.001).

A total of 12/82 (14.8%) PLWH died during admission. People living with HIV who died all had significant HIV-related comorbidities, or other risk factors for COVID-19-related mortality (Table 4). Their hospital stay was shorter than those who survived (median of 3 days vs 7 days), and HIVVL and CD4 counts were not associated with in-hospital mortality in univariate analyses (logrank *P* > 0.05) (Figure 3b & Figure 3d). Creatinine, CRP, PCT and DDIM levels were higher at admission in PLWH who died compared to those who survived (*P* < 0.05 for all comparisons). When compared to HIV-negative patients who died, PLWH who died had higher DDIM levels at admission (2.3 [IQR: 1.6–5.9] vs 1.5 [IQR: 0.9–3.4], MW *P* = 0.05), were younger (mean age 49 vs 64, *T* test *P* = 0.001), less likely to be hypertensive (OR = 0.23, *P* = 0.02) and more likely to have TB (16.7% vs 0.9%, OR = 20.3, *P* = 0.03).

**TABLE 4:** Clinical description of people with HIV who died during admission.

Patient	Age (years)	Gender	Comorbidities	CD4 count (cells/µL)	HIV-1 viral load (IU/mL)	ROX score†
1	60–79	Male	Hypertension	375	LDL	1.9
2	< 40	Female	Overweight	36	LDL	3.0
3	< 40	Female	Active TB‡	106	-	15.6
4	40–59	Male	Diabetes B cell lymphoblastic leukaemia	387	LDL	2.7
5	40–59	Male	CKD stage 5	-	-	-
6	40–59	Female	Suspected SLE§	608	LDL	12.5
7	60–79	Male	CKD stage 5 Epilepsy	159	-	5.7
8	< 40	Male	Chronic HBV HBV viral load = 64 113 IU/mL B cell lymphoma Active TB on treatment at admission	20	3310	10.2
9	60–79	Female	Hypertension Diabetes Obesity CKD stage 3	612	-	3.2
10	40–59	Female	Disseminated CMV CMV viral load = 27 000 IU/mL Cavitating pneumonia	44	1.36 × 10^6^	20.6
11	40–59	Male	Hypertension Diabetes	577	LDL	8.3
12	40–59	Female	Hypertension Diabetes Previous TB	429	LDL	-

ROX, respiratory oxygenation score; CKD, chronic kidney disease; HBV, Hepatitis B virus; CMV, Cytomegalovirus; LDL, lower than detectable level; TB, tuberculosis; SLE, systemic lupus erythematosus.

† , ROX score at admission; ‡, TB treatment started on empirical grounds; §, Anti-nuclear antibody positive, with bicytopenia, rash and joint pain – admitted for haemoptysis and developed nosocomial SARS-CoV-2 infection.

## Discussion

We report a detailed analysis of clinical phenotypes of COVID-19 in hospitalised patients, with and without HIV, and their association with laboratory biomarkers.

People living with HIV had similar levels of COVID-19 severity, whether estimated by levels of supportive care during hospitalisation, ROX scores or laboratory biomarkers when compared to HIV-negative patients. People living with HIV were younger, less likely to be hypertensive, and had lower creatinine levels at admission. People living with HIV with detectable HIVVLs had less severe respiratory disease, with equivalent levels of systemic inflammation as those with suppressed HIVVLs. Similar results have been reported in hospital cohorts from higher-income settings.^7,34^ People living with HIV with suppressed HIVVLs were older, more likely to be on ART, and more likely to have other comorbidities when compared to those with detectable HIVVLs. The prevalence of comorbidities increased with age in this cohort, as has been reported in other studies.^3^ Therefore, it is possible that the association between respiratory disease severity and HIVVL was confounded by baseline differences in age and comorbidities in PLWH, and not necessarily because of HIV viraemia.

Effective ART suppresses viral replication and reverses much of the immunopathology of HIV, but T-cell and monocyte phenotypes, as well as levels of systemic immune activation, remain altered for years afterwards.^21,35^ Monocytes and cytokines involved in monocyte trafficking are central to the pathophysiology of COVID-19.^11^ Monocytes are recruited to tissue by the interaction of their CCR2 receptor and its ligand CCL2, which is dysregulated in severe COVID-19.^9^ HIV infection decreases CCR2 expression on monocytes, and this is reversed with suppressive ART.^21^ Reduced monocyte trafficking to lungs after SARS-CoV-2 infection may reduce later monocyte-derived inflammation in COVID-19, and this may be a mechanism to explain the less severe respiratory disease in PLWH with detectable HIVVLs in this study. Currently reported COVID-19 studies have included few PLWH with detectable HIV VLs, and more research is needed to determine the effect of HIV infection on COVID-19 immune responses.

People living with HIV who died often had significant coexisting comorbidities including active TB infection, lymphoma, disseminated cytomegalovirus, autoimmune disease, or other comorbidities, such as hypertension, diabetes, or CKD. This complicates any discussion of COVID-19 clinical phenotypes in PLWH, who are at risk of opportunistic infections which can cause concomitant respiratory disease and raised inflammatory markers (pulmonary TB or Pneumocystis jirovecii pneumonia [PJP]). Furthermore, without systematic investigation it is difficult to know how much underlying HIV-related disease contributed to in-hospital mortality in this cohort. People living with HIV who died with suppressed viral loads were older, had higher CD4 counts and had additional comorbidities like hypertension, obesity, and diabetes, while those with detectable HIV VLs had significant coinfections or HIV-associated malignancies. The sample size of PLWH who died was small, and it is therefore difficult to draw statistically supported conclusions on these observations. Many of the larger studies which reported that HIV infection is associated with increased risk of severe COVID-19 and related mortality included a higher proportion of men in their analysis than our study.^3,36,37,38^ It is possible that sex and HIV interact with COVID-19 to alter disease phenotypes. Neutrophils play an important role in COVID-19 related immunothrombosis, and neutrophils isolated from women have greater inflammatory responses to interferon, which may allow for better innate immune antiviral response.^39^ Men with severe COVID-19 have altered kynurenic acid metabolism which is associated weaker T-cell responses.^40^ Further research should be undertaken to investigate the interaction of sex, age, and HIV infection on immune responses.

This analysis has several limitations: We analysed records for patients admitted to a single tertiary academic medical centre in an urban area; many patients are sent there by referral which may bias admission towards those with more severe disease. This cohort’s prevalence of HIV infection was within the range for other reported estimates and is likely to be broadly representative of similar hospital cohorts in South Africa.^6,41,42^ Approximately one-third of PLWH in this study did not have HIVVLs measured which may have biased the comparison between detectable versus undetectable HIVVL participants. Strengths of our study include prospective data collection, a validated method for quantifying respiratory disease severity on a continuum, many patients with laboratory biomarkers during admission, and PLWH well characterised in terms of other comorbidities. We have generated several hypotheses related to HIV and COVID-19 for future exploration.

This study shows that PLWH who were hospitalised with COVID-19 did not have significantly different in-hospital mortality rates, levels of inflammatory biomarkers or respiratory disease severity. People living with HIV who died often had other risk factors for COVID-19-associated mortality, or AIDS-defining illness.

## References

[1] Cohen C, Walaza S, Viboud C, et al. Deaths associated with respiratory syncytial and influenza viruses among persons ≥5 years of age in HIV-prevalent area, South Africa, 1998–2009. Emerg Infect Dis J. 2015;21(4):600–608. 10.3201/eid2104.141033 PMC4378466 25811455

[2] Cohen C, Moyes J, Tempia S, et al. Mortality amongst patients with influenza-associated severe acute respiratory illness, South Africa, 2009–2013. PLoS One. 2015;10(3):e0118884. 10.1371/journal.pone.0118884 25786103 PMC4365037

[3] Jassat W, Cohen C, Tempia S, et al. Risk factors for COVID-19-related in-hospital mortality in a high HIV and tuberculosis prevalence setting in South Africa: A cohort study. Lancet HIV. 2021;8(9):E554–E567. 10.1016/S2352-3018(21)00151-X 34363789 PMC8336996

[4] Boulle A, Davies MA, Hussey H, et al. Risk factors for COVID-19 death in a population cohort study from the Western Cape Province, South Africa. Clin Infect Dis. 2020;73(7):e2005–e2015. 10.1093/cid/ciaa1198 PMC7499501 32860699

[5] WHO Global Clinical Platform for COVID-19. Clinical features and prognostic factors of COVID-19 in people living with HIV hospitalized with suspected or confirmed SARS-CoV-2 infection [homepage on the Internet]. World Health Organization; 2021 [cited 2021 Sep 12]. Available from: https://apps.who.int/iris/bitstream/handle/10665/342697/WHO-2019-nCoV-Clinical-HIV-2021.1-eng.pdf

[6] Venturas J, Zamparini J, Shaddock E, et al. Comparison of outcomes in HIV-positive and HIV-negative patients with COVID-19. J Infect. 2021;83(2):217–227. 10.1016/j.jinf.2021.05.020 34051225 PMC8152212

[7] Patel VV, Felsen UR, Fisher M, et al. Clinical outcomes and inflammatory markers by HIV serostatus and viral suppression in a large cohort of patients hospitalized with COVID-19. J Acquir Immune Defic Syndr. 2021;86(2):224–230. 10.1097/QAI.0000000000002578 33433966 PMC8720497

[8] Calligaro GL, Lalla U, Audley G, et al. The utility of high-flow nasal oxygen for severe COVID-19 pneumonia in a resource-constrained setting: A multi-centre prospective observational study. EClinicalMedicine. 2020;28:100570. 10.1016/j.eclinm.2020.100570 33043285 PMC7536126

[9] Consortium Co 19 M omics BAt (COMBAT), Ahern DJ, Ai Z, et al. A blood atlas of COVID-19 defines hallmarks of disease severity and specificity. Cell. 2022;185(5):916–938.e58. 10.1016/j.cell.2022.01.012 35216673 PMC8776501

[10] Thwaites RS, Sanchez Sevilla Uruchurtu A, Siggins MK, et al. Inflammatory profiles across the spectrum of disease reveal a distinct role for GM-CSF in severe COVID-19. Sci Immunol. 2021;6(57):eabg9873. 33692097 10.1126/sciimmunol.abg9873 PMC8128298

[11] Vanderbeke L, Van Mol P, Van Herck Y, et al. Monocyte-driven atypical cytokine storm and aberrant neutrophil activation as key mediators of COVID-19 disease severity. Nat Commun. 2021;12(1):4117. 10.1038/s41467-021-24360-w 34226537 PMC8257697

[12] Hanna SJ, Codd AS, Gea-Mallorqui E, et al. T cell phenotypes in COVID-19 – A living review. Oxford Open Immunol. 2021;2(1):iqaa007. 10.1093/oxfimm/iqaa007 PMC7798577 33575657

[13] Mueller AA, Tamura T, Crowley CP, et al. Inflammatory biomarker trends predict respiratory decline in COVID-19 patients. Cell Rep Med. 2020;1(8):100144. 10.1016/j.xcrm.2020.100144 33163981 PMC7598305

[14] Roca O, Messika J, Caralt B, et al. Predicting success of high-flow nasal cannula in pneumonia patients with hypoxemic respiratory failure: The utility of the ROX index. J Crit Care. 2016;35:200–205. 10.1016/j.jcrc.2016.05.022 27481760

[15] Gottlieb RL, Vaca CE, Paredes R, et al. Early remdesivir to prevent progression to severe Covid-19 in outpatients. N Engl J Med. 2022;386(4):305–315. 10.1056/NEJMoa2116846 34937145 PMC8757570

[16] Hammond J, Leister-Tebbe H, Gardner A, et al. Oral nirmatrelvir for high-risk, nonhospitalized adults with Covid-19. N Engl J Med. 2022;386(15):1397–1408. 10.1056/NEJMoa2118542 35172054 PMC8908851

[17] WHO Solidarity Trial Consortium. Remdesivir and three other drugs for hospitalised patients with COVID-19: Final results of the WHO solidarity randomised trial and updated meta-analyses. Lancet. 2022;399(10339):1941–1953. 10.1016/S0140-6736(22)00519-0 35512728 PMC9060606

[18] Results – Recovery trial [homepage on the Internet]. [cited 2021 Sept 25]. Available from: https://www.recoverytrial.net/results

[19] Moir S, Fauci AS. B cells in HIV infection and disease. Nat Rev Immunol. 2009;9(4):235–245. 10.1038/nri2524 19319142 PMC2779527

[20] Walker B, McMichael A. The T-cell response to HIV. Cold Spring Harb Perspect Med. 2012;2(11):a007054. 10.1101/cshperspect.a007054 23002014 PMC3543107

[21] McCausland MR, Juchnowski SM, Zidar DA, et al. Altered monocyte phenotype in HIV-1 infection tends to normalize with integrase-inhibitor-based antiretroviral therapy. PLoS One. 2015;10(10):e0139474. 10.1371/journal.pone.0139474 26430882 PMC4591977

[22] Riou C, Du Bruyn E, Stek C, et al. Profile of SARS-CoV-2-specific CD4 T cell response: Relationship with disease severity and impact of HIV-1 and active *Mycobacterium tuberculosis* co-infection. Infect Dis (except HIV/AIDS) [serial online]; 2021 [cited 2021 Mar 02]. Available from: http://medrxiv.org/lookup/doi/10.1101/2021.02.16.21251838

[23] Karim F, Gazy I, Cele S, et al. HIV status alters disease severity and immune cell responses in β variant SARS-CoV-2 infection wave [homepage on the Internet]. 2021, p. 20236828. [cited 2021 Sept 27]. Available from: https://www.medrxiv.org/content/10.1101/2020.11.23.20236828v2 10.7554/eLife.67397 PMC8676326 34608862

[24] Snyman J, Hwa SH, Krause R, et al. Similar antibody responses against SARS-CoV-2 in HIV uninfected and infected individuals on antiretroviral therapy during the first South African infection wave. Clin Infect Dis. 2021;75(1):e249–e256. 10.1093/cid/ciab758 PMC8522359 34472583

[25] Barbera LK, Kamis KF, Rowan SE, et al. HIV and COVID-19: Review of clinical course and outcomes. HIV Res Clin Pract. 2021;22(4):102–118. 10.1080/25787489.2021.1975608 34514963 PMC8442751

[26] Kim H, Tanser F, Tomita A, Vandormael A, Cuadros DF. Beyond HIV prevalence: Identifying people living with HIV within underserved areas in South Africa. BMJ Glob Health. 2021;6(4):e004089. 10.1136/bmjgh-2020-004089 PMC8061852 33883186

[27] Simbayi L, Zuma K, Zungu N, et al. South African national HIV prevalence, incidence, behaviour and communication survey, 2017: Towards achieving the UNAIDS 90-90-90 targets [homepage on the Internet]. 2019 [cited 2021 Oct 17]. Available from: https://repository.hsrc.ac.za/handle/20.500.11910/15052

[28] Harris PA, Taylor R, Minor BL, et al. The REDCap consortium: Building an international community of software platform partners. J Biomed Inform. 2019;95:103208. 10.1016/j.jbi.2019.103208 31078660 PMC7254481

[29] RStudio Team. RStudio: Integrated Development for R [homepage on the Internet]. Boston, MA: RStudio, PBC; 2020 [cited 2021 Aug 23]. Available from: http://www.rstudio.com

[30] Kassambara A, Kosinski M, Biecek P. survminer: Drawing survival curves using ‘ggplot2’ [homepage on the Internet]. R package version 048. Available from: https://CRAN.R-project.org/package=survminer

[31] Dardis C. survMisc: Miscellaneous Functions for Survival Data [homepage on the Internet]. 2018. Available from: https://CRAN.R-project.org/package=survMisc

[32] Kassambara A. ggpubr: ‘ggplot2’ Based Publication Ready Plots. R package version 040 [homepage on the Internet]. 2020; Available from: https://CRAN.R-project.org/package=ggpubr

[33] Yoshida K, Bartel A, Chipman JJ, et al. tableone: Create ‘Table 1’ to describe baseline characteristics with or without propensity score weights [homepage on the Internet]. 2021 [cited 2021 Dec 09]. Available from: https://CRAN.R-project.org/package=tableone

[34] Durstenfeld MS, Sun K, Ma Y, et al. Association of HIV infection with outcomes among adults hospitalized with COVID-19. AIDS. 2022;36:391–398. 34750295 10.1097/QAD.0000000000003129 PMC8795492

[35] Cao W, Mehraj V, Kaufmann DE, Li T, Routy JP. Elevation and persistence of CD8 T-cells in HIV infection: The Achilles heel in the ART era. J Int AIDS Soc. 2016;19(1):20697. 10.7448/IAS.19.1.20697 26945343 PMC4779330

[36] Sun J, Patel RC, Zheng Q, et al. COVID-19 disease severity among people with HIV infection or solid organ transplant in the United States: A nationally-representative, Multicenter, Observational Cohort Study [homepage on the Internet]. 2021, p. 21261028. [cited 2021 Oct 20]. Available from: https://www.medrxiv.org/content/10.1101/2021.07.26.21261028v1

[37] Tesoriero JM, Swain CAE, Pierce JL, et al. COVID-19 outcomes among persons living with or without diagnosed HIV infection in New York State. JAMA Netw Open. 2021;4(2):e2037069. 10.1001/jamanetworkopen.2020.37069 33533933 PMC7859843

[38] Nomah DK, Reyes-Urueña J, Díaz Y, et al. Sociodemographic, clinical, and immunological factors associated with SARS-CoV-2 diagnosis and severe COVID-19 outcomes in people living with HIV: A retrospective cohort study. Lancet HIV. 2021;8(11):E701–E710. 10.1016/S2352-3018(21)00240-X 34655549 PMC8514194

[39] Gupta S, Nakabo S, Blanco LP, et al. Sex differences in neutrophil biology modulate response to type I interferons and immunometabolism. Proc Natl Acad Sci U S A. 2020;117(28):16481–16491. 10.1073/pnas.2003603117 32601182 PMC7368314

[40] Cai Y, Kim DJ, Takahashi T, et al. Kynurenic acid may underlie sex-specific immune responses to COVID-19. Sci Signal. 2021;14(690):eabf8483. 10.1126/scisignal.abf8483 34230210 PMC8432948

[41] Mash RJ, Presence-Vollenhoven M, Adeniji A, et al. Evaluation of patient characteristics, management and outcomes for COVID-19 at district hospitals in the Western Cape, South Africa: Descriptive observational study. BMJ Open. 2021;11(1):e047016. 10.1136/bmjopen-2020-047016 PMC7839306 33500292

[42] Parker A, Boloko L, Moolla MS, et al. Clinical features and outcomes of COVID-19 admissions in a population with a high prevalence of HIV and tuberculosis: A multicentre cohort study. BMC Infect Dis. 2022;22(1):559. 10.1186/s12879-022-07519-8 35725387 PMC9207843

